# Common Presence of Phototrophic *Gemmatimonadota* in Temperate Freshwater Lakes

**DOI:** 10.1128/mSystems.01241-20

**Published:** 2021-03-16

**Authors:** Izabela Mujakić, Adrian-Ştefan Andrei, Tanja Shabarova, Lívia Kolesár Fecskeová, Michaela M. Salcher, Kasia Piwosz, Rohit Ghai, Michal Koblížek

**Affiliations:** a Laboratory of Anoxygenic Phototrophs, Centre Algatech, Institute of Microbiology of the Czech Academy of Sciences, Třeboň, Czechia; b Department of Ecosystem Biology, Faculty of Science, University of South Bohemia, České Budějovice, Czechia; c Department of Aquatic Microbial Ecology, Institute of Hydrobiology, Biology Centre of the Czech Academy of Sciences, České Budějovice, Czechia; University of British Columbia

**Keywords:** *Gemmatimonadota*, *Gemmatimonadetes*, anoxygenic phototrophs, aquatic bacteria, MAGs, metagenome, photosynthesis gene cluster, freshwater ecology, CARD-FISH

## Abstract

Members of the bacterial phylum *Gemmatimonadota* are ubiquitous in most natural environments and represent one of the top 10 most abundant bacterial phyla in soil. Sequences affiliated with *Gemmatimonadota* were also reported from diverse aquatic habitats; however, it remains unknown whether they are native organisms or represent bacteria passively transported from sediment or soil. To address this question, we analyzed metagenomes constructed from five freshwater lakes in central Europe. Based on the 16S rRNA gene frequency, *Gemmatimonadota* represented from 0.02 to 0.6% of all bacteria in the epilimnion and between 0.1 and 1% in the hypolimnion. These proportions were independently confirmed using catalyzed reporter deposition-fluorescence *in situ* hybridization (CARD-FISH). Some cells in the epilimnion were attached to diatoms (*Fragilaria* sp.) or cyanobacteria (*Microcystis* sp.), which suggests a close association with phytoplankton. In addition, we reconstructed 45 metagenome-assembled genomes (MAGs) related to *Gemmatimonadota*. They represent several novel lineages, which persist in the studied lakes during the seasons. Three lineages contained photosynthesis gene clusters. One of these lineages was related to Gemmatimonas phototrophica and represented the majority of *Gemmatimonadota* retrieved from the lakes’ epilimnion. The other two lineages came from hypolimnion and probably represented novel photoheterotrophic genera. None of these phototrophic MAGs contained genes for carbon fixation. Since most of the identified MAGs were present during the whole year and cells associated with phytoplankton were observed, we conclude that they represent truly limnic *Gemmatimonadota* distinct from the previously described species isolated from soils or sediments.

**IMPORTANCE** Photoheterotrophic bacterial phyla such as *Gemmatimonadota* are key components of many natural environments. Its first photoheterotrophic cultured member, Gemmatimonas phototrophica, was isolated in 2014 from a shallow lake in the Gobi Desert. It contains a unique type of photosynthetic complex encoded by a set of genes which were likely received via horizontal transfer from *Proteobacteria*. We were intrigued to discover how widespread this group is in the natural environment. In the presented study, we analyzed 45 metagenome-assembled genomes (MAGs) that were obtained from five freshwater lakes in Switzerland and Czechia. Interestingly, it was found that phototrophic *Gemmatimonadota* are relatively common in euphotic zones of the studied lakes, whereas heterotrophic *Gemmatimonadota* prevail in deeper waters. Moreover, our analysis of the MAGs documented that these freshwater species contain almost the same set of photosynthesis genes identified before in Gemmatimonas phototrophica originating from the Gobi Desert.

## INTRODUCTION

Microorganisms conduct key biogeochemical processes involved in the main fluxes of matter and energy on Earth. Most microbial diversity remains uncultured, and only analyses of environmental DNA samples have made it possible to unravel existing microbial diversity and to identify the main species involved (1). Indeed, out of 112 known bacterial phyla in the Genome Taxonomy Database (GTDB) (2), more than one half are still recognized only from environmental sequences (3).

One of the phyla that was first identified using molecular phylogenetic methods was *Gemmatimonadota* (also called *Gemmatimonadetes* [4]), which was originally established as the so-called BD group based on five 16S rRNA gene sequences, which originated from deep-sea sediments, soils, and reactor sludge (5, 6). Independently, the group was also proposed as a candidate division KS-B based on three 16S rRNA gene sequences retrieved from coastal sediment samples from French Guiana (7).

The first cultured strain T27 belonging to the BD/KS-B group was isolated from a wastewater treatment plant in Japan. The isolate was named Gemmatimonas aurantiaca and established the new phylum *Gemmatimonadota*, along with its first class *Gemmatimonadetes* and genus *Gemmatimonas* (8). Subsequently, three more *Gemmatimonadota* genera (*Gemmatirosa*, *Longimicrobium*, and *Roseisolibacter*) with type strains were described from various soil environments (9–11). Apart from class *Gemmatimonadetes*, phylum *Gemmatimonadota* consists of four more class-level groups which include class *Longimicrobia*, two terrestrial groups (BD2-11 and S0134), and one marine benthic group (PAUC43f) (10, 12). The four cultured representatives from the phyla were all chemo-organoheterotrophs that require oxygen and grow under fully aerobic or semiaerobic conditions (8–11). An interesting metabolic potential and ecological role was reported for G. aurantiaca, as this species has the ability to reduce the greenhouse gas N_2_O (13). However, with the discovery of Gemmatimonas phototrophica, which contains photosynthetic reaction centers (14, 15), *Gemmatimonadota* were added to several bacterial phyla containing anoxygenic phototrophic species alongside *Proteobacteria*, *Chlorobi* (now included as a class-level lineage in *Bacteroidota* [2]), *Chloroflexota*, *Firmicutes* (*Bacillota*), *Acidobacteriota*, and the newly discovered phylum “*Candidatus* Eremiobacterota” (WPS-2) (14, 16, 17). Anoxygenic phototrophs, such as G. phototrophica, are able to support their metabolism by harvesting light using bacteriochlorophylls; however, they require a supply of organic substrate for growth (18). Another characteristic found in *G. phototrophica* is the organization of its photosynthesis genes into a cluster called the photosynthesis gene cluster (PGC). Interestingly, the gene arrangement in the PGC of *G. phototrophica* is very similar to the one found in *Proteobacteria*, so it has been suggested that phototrophy in *Gemmatimonadota* originates from an ancient horizontal gene transfer of the entire PGC from *Proteobacteria* (14). As yet, this is the only known case of horizontal transfer of an entire set of photosynthesis genes between distant bacterial phyla (14, 19).

Information about the prevalence of *Gemmatimonadota* in different habitats is continuously growing, although information about their ecology is scarce. Members of this phylum were found in many natural environments (12, 20–22) and represent the eighth most abundant phylum in soils, accounting for about 1 to 2% of bacteria in soils worldwide (23). Their highest contributions are typically found in fertile agricultural and forest soils (20) but are also present in more unique soil environments, such as arid Antarctic Dry Valley soils (24, 25). It has been suggested that *Gemmatimonadota* may be relatively more abundant in dry soils (26). On the other hand, from the available data, it is known that they are also present in aquatic environments, such as freshwater lakes (27), sediments (22, 28–30), and estuaries (31, 32). In addition, *G. phototrophica* was isolated from a freshwater lake in Inner Mongolia (18). However, this organism does not grow in liquid culture and requires microaerophilic conditions, which are more typical for sediment-dwelling species. Thus, the data showing that *Gemmatimonadota* prefer dry environments does not seem to be universal. There is probably a large ecological and functional diversity among the members of *Gemmatimonadota*. The question remains whether *Gemmatimonadota*-related sequences identified in lakes originate from strictly limnic species, or perhaps they are just a passive component that enters the lakes along with runoff waters from surrounding soil.

Current progress in sequencing technologies and bioinformatics has circumvented the necessity for cultivated representatives and allowed biological and ecological inferences to be drawn by using genomic data recovered directly from microbial communities. Over the past few years, the usage of metagenome-assembled genomes (MAGs) has allowed the description of many novel bacterial divisions and unearthed large radiations in the prokaryotic tree of life (33). This approach has already led to the discovery of new phototrophic organisms belonging to the yet uncultured candidate phylum “*Ca*. Eremiobacterota” (16, 34). Also, two MAGs belonging to *Gemmatimonadota* were recovered from Lake Baikal. One MAG was more similar to Gemmatirosa kalamazoonensis found in soils and seemed more abundant at a depth of 5 m. The other MAG-encoded rhodopsin gene and was closely related to the phototrophic species *G. phototrophica* and showed a higher abundance at 20 m (35).

Therefore, in order to address the question whether there are any truly limnic *Gemmatimonadota* and to investigate their diversity, we analyzed metagenome data from five freshwater lakes in Czechia and Switzerland. The lakes were chosen based on their trophic status and included a representative mesoeutrophic Římov Reservoir, a dystrophic Jiřická pond, oligomesotrophic Lake Zurich and Lake Constance, and an ultraoligotrophic Lake Thun (Table 1). The metagenome sequences were collected over several years and seasons. The reconstructed MAGs were analyzed with the aim to identify the most common freshwater and photoheterotrophic *Gemmatimonadota* and to analyze their spatiotemporal variability. Using catalyzed reporter deposition-fluorescence *in situ* hybridization (CARD-FISH), cells of *Gemmatimonadota* were visualized for the first time in their natural environment, and their association with other organisms was observed.

**TABLE 1:** Basic characteristics of the lakes studied

Characteristic	Římov Reservoir	Jiřická pond	Lake Zurich	Lake Constance	Lake Thun
Country^*a*^	CZ	CZ	CH	CH	CH
Latitude	48.50°N	48°36'N	47°18'N	47°32'N	46°41′N
Longitude	14.29°E	14°40'E	8°34’E	9°31'E	7°43′E
Altitude (m)	470	892	406	395	558
Area (km^2^)	2.10	0.035	88.66	536	48.3
Volume (m^3^)	34.5 × 10^6^	6.59 × 10^3^	3.3 × 10^9^	48 × 10^9^	6.5 × 10^9^
Avg depth (m)	16.5	1.8	49	90	136
Maximum depth (m)	43	4.5	136	251	217
Mean hydraulic residence time	77 days	9 days	1.4 yrs	5 yrs	1.8 yrs
Trophic status	Mesoeutrophic	Dystrophic	Oligomesotrophic	Oligomesotrophic	Ultraoligotrophic
Mixing type	Dimictic	Polymictic	Monomictic	Monomictic	Monomictic
Sampling	2015−2017	2016−2017	2010−2019	2018	2018
No. of samples^*b*^	E = 10, H = 8	E = 5	E = 6, H = 4	E = 2, H = 2	E = 1, H = 1

a CZ, Czechia; CH, Switzerland.

b E refers to epilimnion, and H refers to hypolimnion. The exact depths for each of the lakes are provided in Materials and Methods.

## RESULTS

### Abundance of *Gemmatimonadota*.

The presence of *Gemmatimonadota* in freshwater lakes was first assessed by using relative 16S rRNA abundances extracted from our metagenomic data sets. In all studied lakes, *Gemmatimonadota* formed only a small part of the bacterial community with relative abundances typically below 1%. The highest relative abundance was found in Římov Reservoir, where *Gemmatimonadota* sequences were present over the entire sampling period (2015 to 2017). Interestingly, their relative abundance was higher in the hypolimnion (0.58% ± 0.23%; *n *= 8) than in the epilimnion (0.24% ± 0.21%; *n *= 10), with the highest numbers (1.03%) in August 2016. A similar pattern of higher contribution in the hypolimnion (0.44% ± 0.27%; *n *= 4) than in the epilimnion (0.28% ± 0.25%; *n *= 6) was also observed in Lake Zurich, with the highest relative abundance occurring in spring (13 May 2013, 0.77%). The general pattern of higher abundance in the hypolimnion was observed also in both Lake Constance and Lake Thun and was recorded in summer with 0.81% and 0.58% relative abundance, respectively. Finally, the lowest contribution of *Gemmatimonadota* was found in Jiřická pond, where they represented less than 0.1% of the prokaryotic community, with a maximum of 0.09% recorded in summer, in August 2017.

Statistical analysis using distance-based linear models (DistLM) showed that the only environmental factor driving the *Gemmatimonadota* community in Římov Reservoir was water temperature, which explained 47% of the variability in the data set (*P* = 0.0002, pseudo-*F* = 13.303). A separate analysis of epilimnion and hypolimnion samples showed that temperature was also important in epilimnion (*P* = 0.0079, pseudo-*F* = 5.7106, and 41.6% of the explained variability in data set) but not in hypolimnion.

### Metagenome-assembled genomes of *Gemmatimonadetes* and their distribution.

To explore the diversity of aquatic *Gemmatimonadota*, we performed genome-resolved metagenomics analyses. The freshwater MAGs (*n *= 45) obtained in our study were analyzed together with those publicly available (*n *= 226) in March 2019 (see Table S1 in the supplemental material for a complete list). Most of the MAGs assembled by us were obtained from Římov Reservoir (*n* = 27), followed by Lake Zurich (*n* = 10), Lake Constance (*n* = 5), Lake Thun (*n* = 2), and Jiřická pond (*n* = 1). Publicly available MAGs originated from various environments: mostly from marine habitats (*n* = 36) and soils (*n* = 37), followed by sediments of soda lakes (*n* = 38), permafrost (*n* = 23), and other (*n* = 11). Only three MAGs from freshwater environments were available in the database at the time of the study.

10.1128/mSystems.01241-20.6 TABLE S1 List of all MAGs used in phylogenomic analyses, consisting of freshwater MAGs assembled from five freshwater lakes as well as MAGs downloaded from GTDB. The first sheet presents an overview of all genomes, the second sheet shows detailed information about the downloaded genomes, the third sheet contains accession numbers of the freshwater *Gemmatimonadota* MAGs, and the fourth sheet contains CheckM output and summary information for each MAG. Download Table S1, XLSX file, 0.04 MB. Copyright © 2021 Mujakić et al. 2021 Mujakić et al. https://creativecommons.org/licenses/by/4.0/ This content is distributed under the terms of the Creative Commons Attribution 4.0 International license.

**FIG 1:**
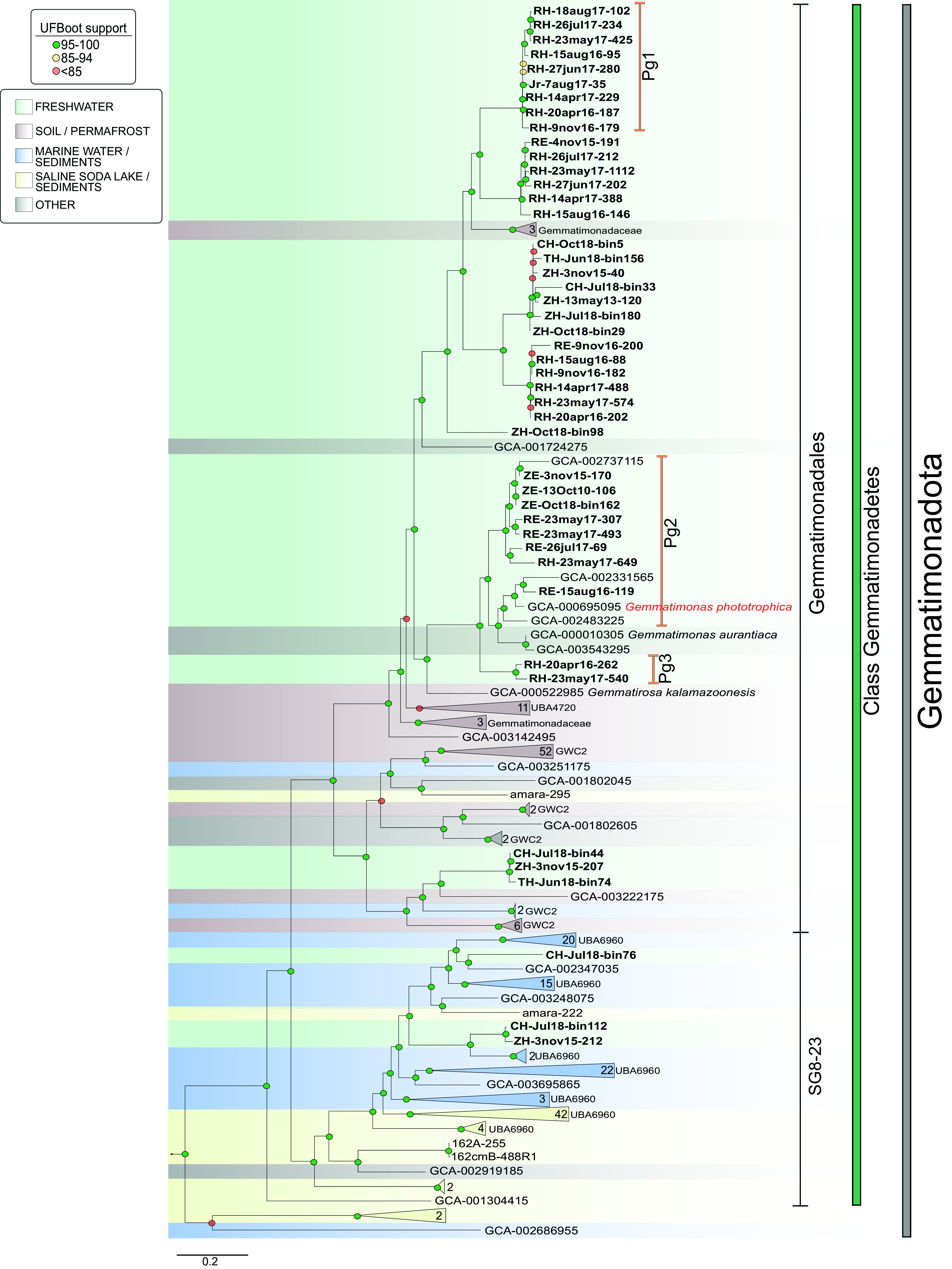
Maximum-likelihood tree (1,000 ultrafast bootstrap replicates, 121 conserved concatenated marker proteins) showing 45 MAGs of freshwater *Gemmatimonadota* as well as 226 MAGs and genomes of cultures collected from GTDB originating from different environments. The different environments are shown by different colors (left legend). The numbers shown at collapsed branches (i.e., 11 and 52) indicate the numbers of genomes (not shown) comprising the respective taxonomic categories. Genomes belonging to *Fibrobacterota* were used as an outgroup to root the tree. The annotations Pg1 to Pg3 define groups of photoheterotrophic *Gemmatimonadota*. Order, class-level, and phylum taxonomic labels are indicated through vertical delimiters (right part of the figure). The strength of support for internal nodes (assessed by ultrafast bootstrapping) is shown through colored circles (left legend). Details on all genomes can be found in Table S1 in the supplemental material.

Phylogenomically, based on Genome Taxonomy Database (GTDB) taxonomy (39), all MAGs recovered in this study clustered within class *Gemmatimonadetes* (Fig. 1). Although they were all recovered from a freshwater environment, six of them were more closely related to publicly available MAGs from environments like soils and sediments. MAGs CH-Jul18-bin44, ZH-3nov15-207, and TH-Jun18-bin75 clustered with MAGs from soils, and CH-Jul18-bin76, CH-Jul18-bin112, and ZH-3nov15-212 clustered with MAGs from sediments. Similarly, in 16S rRNA phylogeny (see Fig. S1 in the supplemental material), all freshwater MAGs with recovered 16S rRNA genes clustered within class *Gemmatimonadetes*, with the exception of two previously mentioned MAGs (CH-Jul18-bin112 and ZH-3nov15-212) that clustered within the BD2-11 terrestrial group based on SILVA SSU v138 database taxonomy (12).

10.1128/mSystems.01241-20.1 FIG S1 Maximum-likelihood phylogeny of 16S rRNA genes of freshwater *Gemmatimonadota* as well as reference sequences recovered from SILVA SSU v138 database (Ref NR 99; length > 1,200 bp). The numbers shown at collapsed branches (i.e., 105 and 154) indicate the numbers of 16S rRNA gene sequences (not shown) comprising the respective taxonomic categories. Photoheterotrophic *Gemmatimonadota* are marked with orange, and the rest of the freshwater MAGs are marked with green. Class-level taxonomic labels based on SILVA database taxonomy are indicated through vertical delimiters (right part of the figure). Labels for six newly defined clusters (five from class *Gemmatimonadetes* and one from BD2-11 terrestrial group) are indicated in the legend on the left part of the figure. Several genomes appertaining to *Cyanobacteria* and *Fibrobacterota* were used as outgroups to root the tree. The strength of support for internal nodes (assessed by ultrafast bootstrapping) is shown through colored circles (left legend). Download FIG S1, PDF file, 0.3 MB. Copyright © 2021 Mujakić et al. 2021 Mujakić et al. https://creativecommons.org/licenses/by/4.0/ This content is distributed under the terms of the Creative Commons Attribution 4.0 International license.

### 16S rRNA gene diversity.

**FIG 2:**
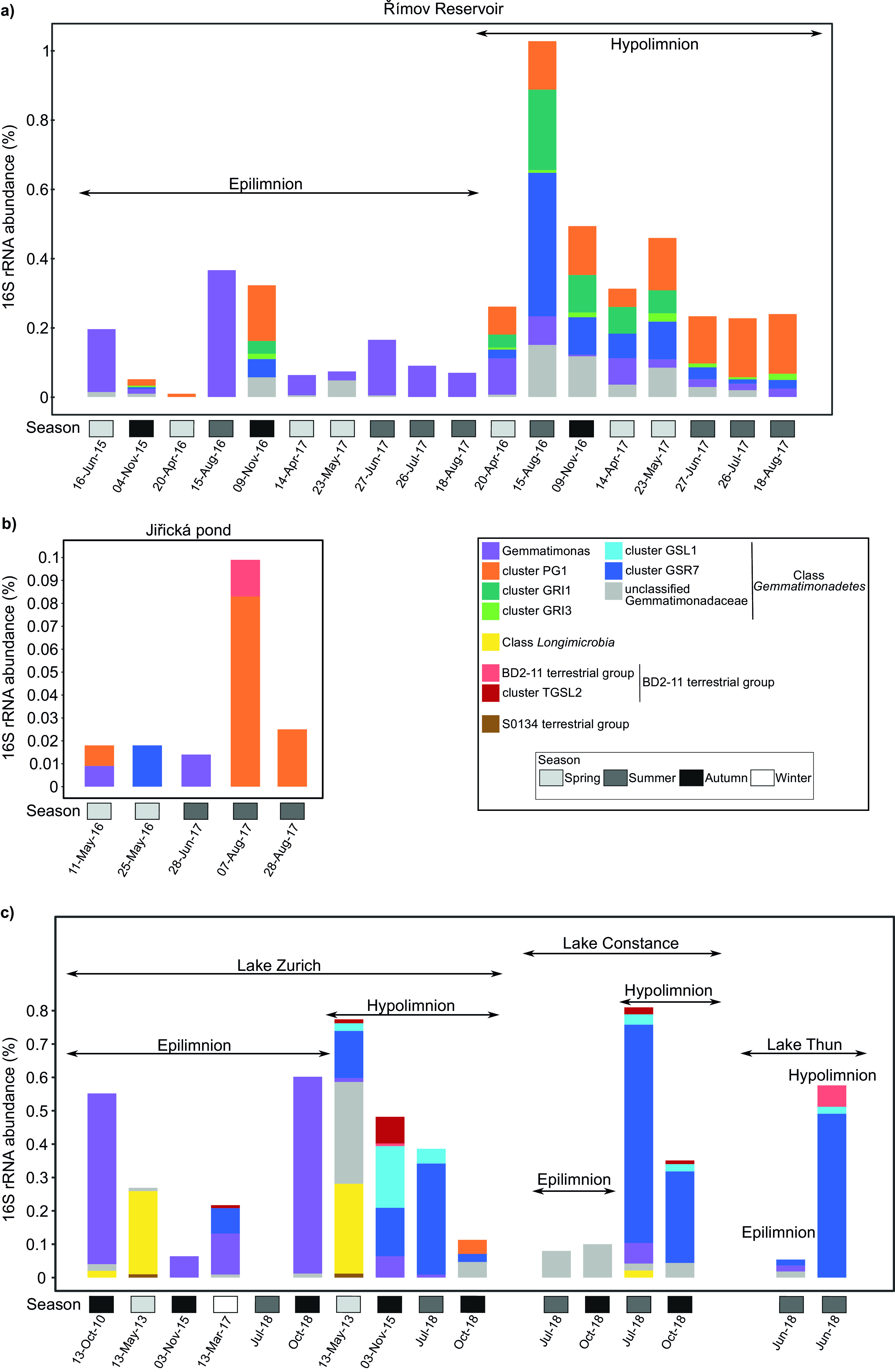
Relative abundance of *Gemmatimonadota* based on 16S rRNA gene fragments from metagenomes of five contrasting freshwater environments. These five freshwater environments are as follows: Římov Reservoir (a), Jiřická pond (b), and Lake Zurich, Lake Constance, and Lake Thun (c). The figure depicts the classification of 16S rRNA gene fragments (as unassembled shotgun reads) retrieved from five freshwater data sets. The *x* axis shows the sampling dates, while the *y* axis indicates the percentage of *Gemmatimonadota* within the prokaryotic communities. The sample collection time, following a four-season breakdown, is indicated by the gray-scale-colored boxes arranged along the *x* axis.

The 16S rRNA gene fragments from metagenomes were classified taxonomically. As most of the *Gemmatimonadota* phylum consists of environmental sequences that remain uncultured, many of the fragments could not be classified. Therefore, in order to increase taxonomic resolution of unclassified *Gemmatimonadota*, we have used 16S rRNA gene sequences from our MAGs (Fig. S1) to define six new clusters (five from class *Gemmatimonadetes* and one from BD2-11 terrestrial group). In Římov Reservoir, the *Gemmatimonadota* community was solely composed of the class *Gemmatimonadetes* (Fig. 2). The genus *Gemmatimonas* was present in all seasons and both depths, but it was more abundant in the epilimnion, especially in summer (15-Aug-16). Epilimnion was dominated by the genus *Gemmatimonas* and unclassified *Gemmatimonadaceae*, with the exception of three samples (two in November and one in April) where other clusters were also present. In contrast, hypolimnion samples contained several different clusters, including phototrophic cluster PG1 that was one of the dominant clusters in every season. Similarly, clusters GRI1 and GSR7 were predominantly present in the hypolimnion. The presence of these three clusters in the epilimnion occurred only in November and April, when the reservoir was mixed. In Jiřícká pond, the *Gemmatimonadota* community was composed of three clusters from the class *Gemmatimonadetes*, i.e., genus *Gemmatimonas*, cluster GSR7, and cluster PG1, the latter being mostly dominant in summer. Additionally, the BD2-11 terrestrial group was also present in low proportions in summer. The highest taxonomic diversity within *Gemmatimonadota* was observed in Swiss lakes, where three other class-level groups in addition to *Gemmatimonadetes* were recovered, i.e., representatives of classes *Longimicrobia*, BD2-11 and S0134, groups that were previously known only from terrestrial 16S rRNA gene sequences (12). Here, genus *Gemmatimonas* was also predominantly present in the epilimnion, whereas cluster GSR7 dominated the hypolimnion. In Lake Zurich, a large contribution of class *Longimicrobia* was recorded in a spring sample (13-May-13) in both the epilimnion and hypolimnion.

### Photosynthesis genes in *Gemmatimonadota*.

Further analyses of the assembled genomes of *Gemmatimonadota* revealed that 19 out of 45 freshwater MAGs contained phototrophic genes. In the phylogenetic tree, we could differentiate them into three distinct phototrophic groups (Fig. 1). Phototrophic group 1 contained eight MAGs from the hypolimnion of Římov Reservoir and one from Jiřická pond. Phototrophic group 2 was formed of nine MAGs (three from the epilimnion of Lake Zurich, five from the epilimnion of Římov Reservoir, and one from the hypolimnion of Římov Reservoir). Phototrophic group 2 also included the only cultured photoheterotrophic strain of the phylum, *G. phototrophica*, as well as the only three MAGs from freshwater environments that were publicly available. Phototrophic group 3 contained two MAGs from the hypolimnion of Římov Reservoir. All these MAGs were recovered from different time points (several years and different seasons).

**FIG 3:**
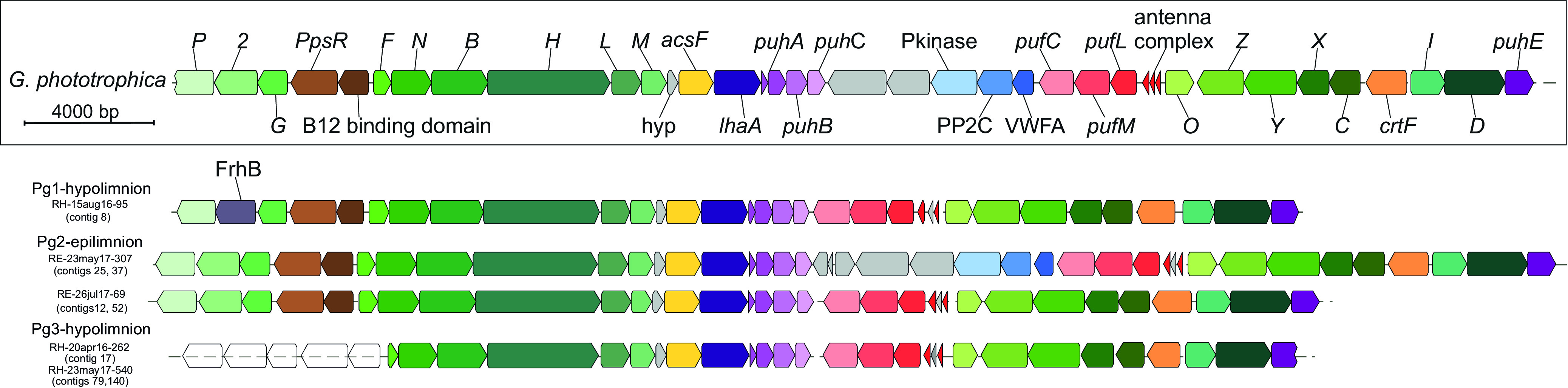
Structure and composition of photosynthetic gene clusters of Gemmatimonas phototrophica (top) and phototrophic MAGs of *Gemmatimonadota* that clustered into three photoheterotrophic groups. The names of the MAGs used to depict each photosynthetic group (photosynthetic group 1 [Pg1] to Pg3) are shown at the right part of the figure. *bch* genes involved in bacteriochlorophyll biosynthesis (green), *puf* operon encoding the reaction center (red), *puh* operon (pink/purple), carotenoid biosynthesis genes (orange), *ppsR* gene and B12 binding domain (brown), *acsF* (yellow), *lhaA* (dark purple), genes not involved in photosynthesis (blue), and hypothetical genes (gray) are indicated. A list of all genes is provided in Table S4.

Using representatives from each of these groups, we reconstructed the photosynthetic gene cluster and compared its structure and organization with the PGC found in *G. phototrophica* (14). For phototrophic groups 1 and 2, we reconstructed the entire PGC. In the case of phototrophic group 3, the PGC was missing several genes due to the incompleteness of the genomes (MAGs had completeness of 71.07. and 83.16%). From our findings, it appears that limnic *Gemmatimonadetes* have the same or very similar organization of phototrophic genes as *G. phototrophica* AP64 (Fig. 3 and Table S4). A distinct trait of *G. phototrophica* is a fragmented PGC divided by a set of hypothetical genes. The same split PGC was observed in three out of nine MAGs from phototrophic group 2, but it was absent in phototrophic groups 1 and 3. Furthermore, we observed the presence of the gene *frhB* (coenzyme F420-reducing hydrogenase, beta subunit) in the operon *bchP2G*, in the PGC of all members of phototrophic group 1, at the position where all other phototrophic *Gemmatimonadetes* have *bch2*. A full list of phototrophic genes for each MAG, as well as their distribution on contigs, is provided as Table S4.

As the presence of ribulose 1,5-bisphosphate carboxylase enzyme (RuBisCO) genes was previously reported in some sediment *Gemmatimonadota* MAGs, we also searched for them in our freshwater MAGs. We identified type IV RubisCO-like genes in eight phototrophic freshwater MAGs from Římov Reservoir (Fig. S3). Seven of these MAGs belonged to phototrophic group 1 recovered from hypolimnion, and one to phototrophic group 2 recovered from the epilimnion of Římov Reservoir.

### Average amino acid identities and 16S rRNA and photosynthetic gene similarities of photoheterotrophic *Gemmatimonadetes*.

We calculated average amino acid identities (AAI) between all the freshwater MAGs and the three genomes of cultured reference strains (Gemmatimonas phototrophica, Gemmatimonas aurantiaca, and *Gemmatirosa kalamazoonesis*) (Fig. S4). All MAGs of the phototrophic group 1 represent the same species, with AAI values of 99 and 100%, that was recovered in both Římov Reservoir and Jiřická pond, multiple times through different years and seasons. When phototrophic group 1 was compared with phototrophic groups 2 and 3, AAI values were below 65%, suggesting that these groups represent different genera. Similarly, phototrophic group 1 showed AAI values below 65% (specifically 55 and 56%) compared with cultured representatives. In phototrophic group 2, the three MAGs from Lake Zurich represent the same species (AAI, 95 to 100%) recovered in different years, whereas other MAGs from Římov Reservoir in this group seem to belong to the same genus but are all different species (AAI, 77 to 82%). Phototrophic group 3 consists of two MAGs that represent the same species (AAI, 96 and 100%). Both phototrophic groups 2 and 3 (AAI, 66 to 68%) belong to the same genus as the cultured representatives *G. phototrophica* and *G. aurantiaca* (AAI 66%) but are different from *Gemmatirosa kalamazoonensis* (AAI, 56%).

From the phototrophic MAGs (phototrophic group 1), one MAG from the hypolimnion of Římov Reservoir (RH-18aug17-102) contained a 16S rRNA gene, which showed 92.8% identity with *G. phototrophica* and 91.5% and 89.6% identity with *G. aurantiaca* and *Gemmatirosa kalamazoonensis*, respectively (Fig. S1), and one MAG from Jiřická pond (Jr-7aug17-35) showed 92.1% 16S rRNA gene sequence identity with *G. phototrophica*.

To further analyze the differences among the identified phototrophic groups, we compared the *pufM* and *acsF* gene sequence identities between them and type species *G. phototrophica*. Both of these genes are markers for anoxygenic photoheterotrophic bacteria (22, 40). For the *pufM* gene (encoding the M subunit of the bacterial photosynthetic reaction center), phototrophic group 2 showed only 80 to 85% identity with *G. phototrophica*. Much lower identities were found for hypolimnion groups 3 and 1 (77 to 80% and 72%, respectively). The *acsF* gene (aerobic oxidative cyclase gene) gave similar results with only 71% identity between phototrophic group 1 and *G. phototrophica*. MAGs from phototrophic groups 2 and 3 had 79 to 89% and 78% identity of the *acsF* gene of *G. phototrophica*, respectively.

### Relative abundance of MAGs of phototrophic and nonphototrophic *Gemmatimonadota*.

**FIG 4:**
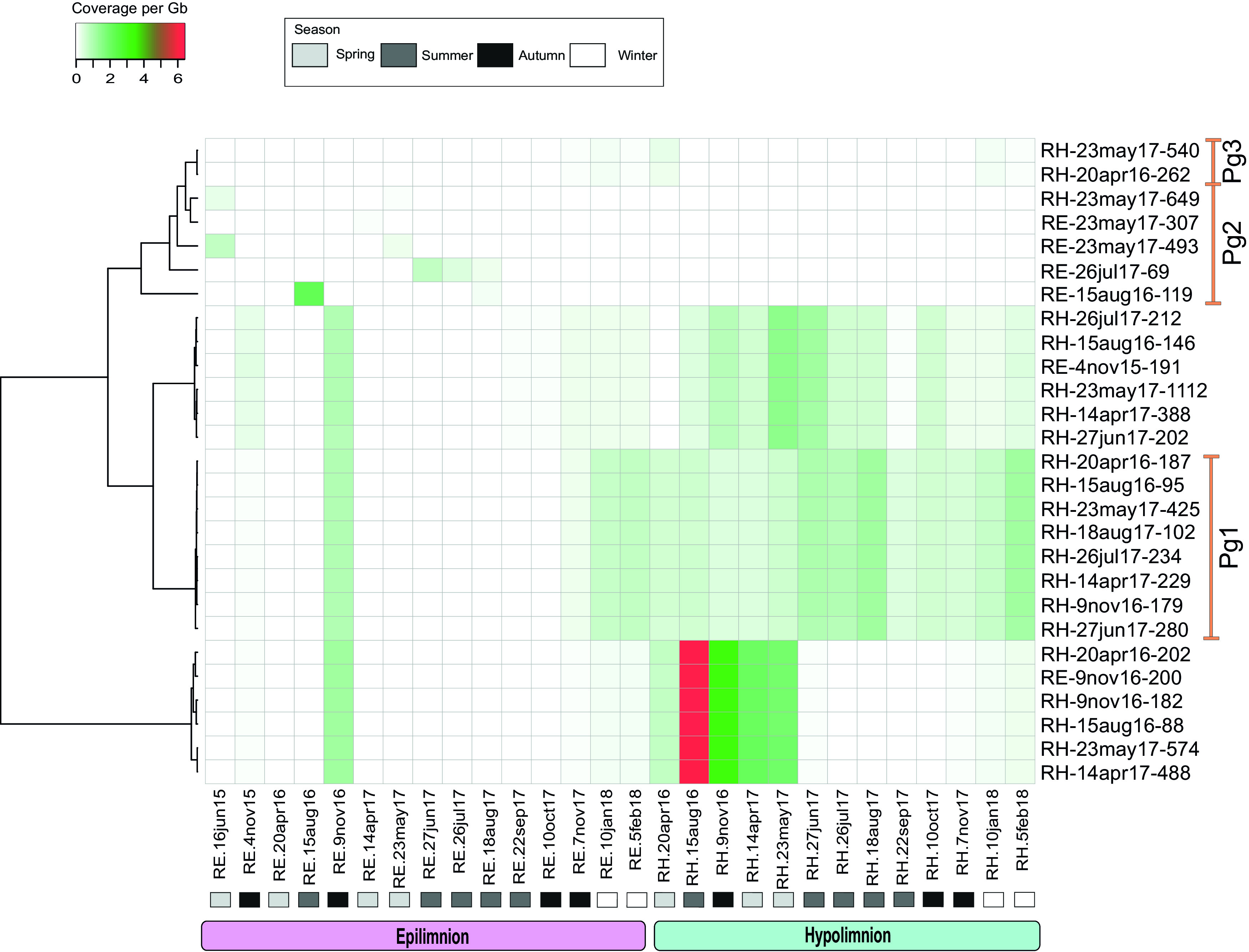
Heatmap of the relative abundance of 27 *Gemmatimonadota* MAGs gained from Římov Reservoir in 28 metagenomes from Římov Reservoir (Table S3). MAGs are clustered using complete linkage and Euclidian correlation. Columns are labeled with the sampling date, the depth of the sample (RE = epilimnion [0.5 m] and RH = hypolimnion [30 m]) and season. Three groups of MAGs (Pg1 to Pg3) containing photosynthetic gene clusters representing photoheterotrophic *Gemmatimonadota* are labeled with orange lines. Color key of abundance (coverage per gigabase) is shown in the top left corner, with red indicating the highest abundance and white indicating that a MAG is not present in a metagenome.

In order to compare the relative abundance of phototrophic and nonphototrophic *Gemmatimonadota*, we used 28 metagenomes from Římov Reservoir from which 27 MAGs were recovered for fragment recruitment (Fig. 4). Nonphotosynthetic MAGs showed the highest contribution in the hypolimnion, especially in summer (15 August 2016); however, their abundance was not constant and changed over time. Similarly, photosynthetic group 1 was also present mostly in the hypolimnion, and its relative abundance varied during the season. The only occurrence of this group in the epilimnion was in late autumn (9 November 2016), at times of deep mixing of the reservoir (41). In contrast, photosynthetic groups 2 and 3 were present mostly in the epilimnion but remained at very low relative abundances in all seasons and at all depths.

### CARD-FISH analyses.

**FIG 5:**
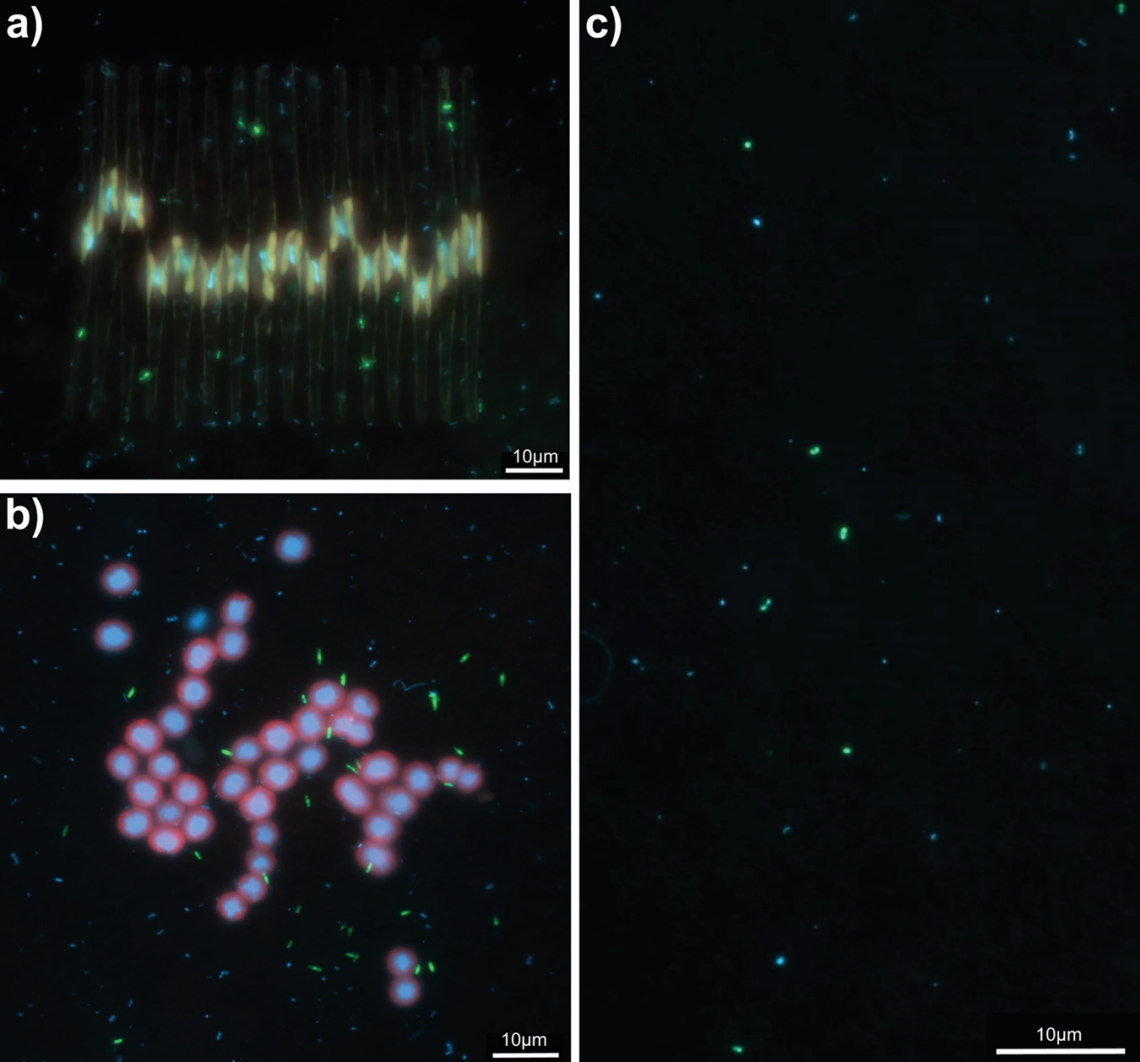
Overlay images of *Gemmatimonadetes* cells in Římov Reservoir visualized by CARD-FISH with probe Gemma_801. (a) *Gemmatimonadetes* associated with a *Microcystis* sp. colony. (b) *Gemmatimonadetes* observed on a colony of *Fragilaria* sp. (c) Free-living *Gemmatimonadetes* observed in the hypolimnion of Římov Reservoir. The probe signal is displayed in green, DAPI staining is displayed in blue, and autofluorescence of *Cyanobacteria* is displayed in red.

**FIG 6:**
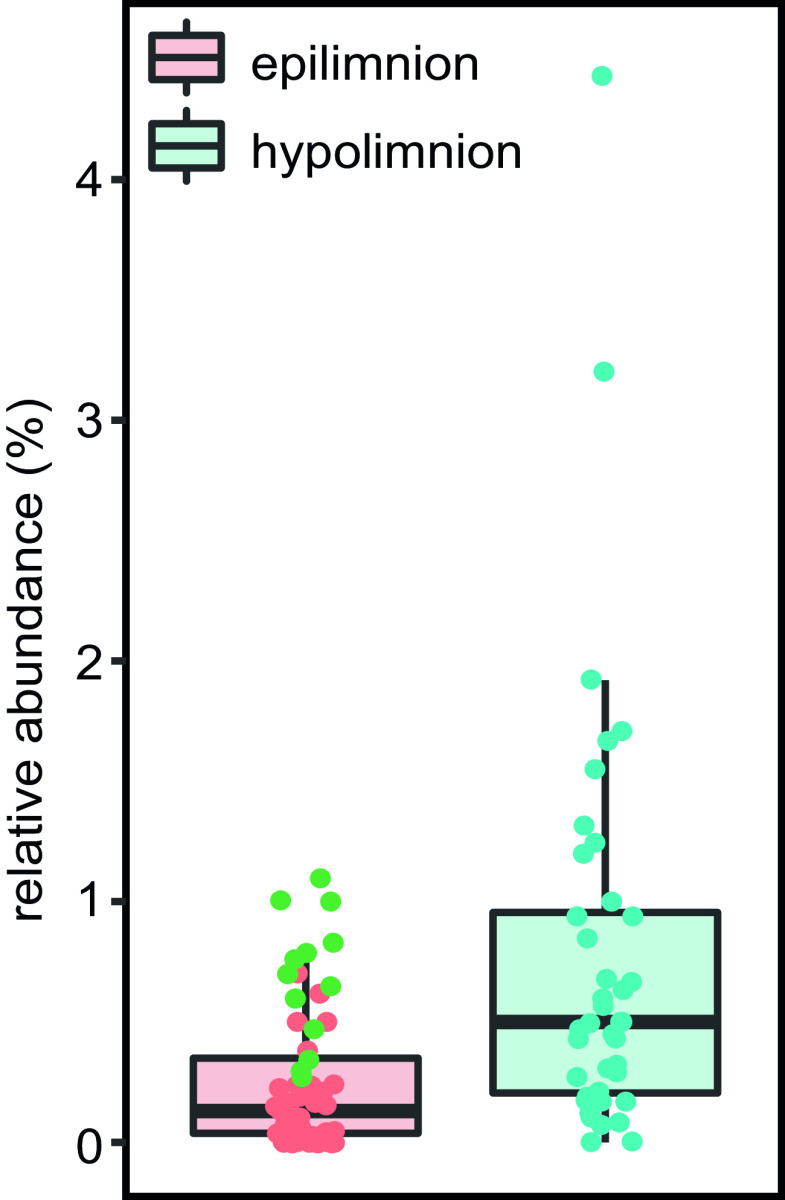
Relative abundance of planktonic *Gemmatimonadetes* in the epi- and hypolimnion of Římov Reservoir obtained by CARD-FISH analyses with the probe Gemma_801. Epilimnion samples where *Fragilaria* sp. and *Microcystis* sp. were observed are colored in green. The difference between the epilimnion and hypolimnion is significant (Mann-Whitney U test, [*P =* <0.001]).

In order to visualize members of the class *Gemmatimonadetes* and access more information on their distribution and potential associations within freshwater environments, we designed the CARD-FISH probe Gemma_801 and applied it to a set of samples obtained from Římov Reservoir, comprising eight longitudinal transects collected during summer 2015. The hybridized cells were also counted; however, since the probe *in silico* matches only 35% of all *Gemmatimonadetes*, the presented numbers, while corresponding with 16S rRNA gene abundance from metagenomes, reflect only the detected, and not total *Gemmatimonadetes*, implying that the observed numbers can underestimate the absolute abundance of *Gemmatimonadetes* in the environments studied. The lowest relative (mean = 0.09%, minimum [min] = 0.00, maximum [max] = 0.19%) and absolute (mean = 3.25 × 10^3^ cells ml^−1^, min = 0.00, max = 7.9 × 10^3^ cells ml^−1^) abundances were observed at the river station of the reservoir (Fig. S2). However, at stations 2 and 3 (stations following the “river station” in the longitudinal transect), the contribution of hybridized planktonic cells had already reached up to 1.0% (4.3 × 10^4^ cells ml^−1^) during a bloom of *Fragilaria* sp. at the end of June. Besides free-living *Gemmatimonadetes*, we observed hybridized cells that seemed to be attached to diatoms (Fig. 5a). On some *Fragilaria* sp. colonies, hybridized *Gemmatimonadetes* contributed up to 22% of all bacterial cells detected on the diatom surface. In addition, *Gemmatimonadetes* seemed to be associated with cyanobacteria, as higher densities were detected with agglomerations of *Microcystis* sp. colonies (Fig. 5b and Fig. S5a). However, the highest numbers of hybridized cells were observed in the hypolimnion (≥10 m) of Římov Reservoir (up to 4.43% and 2.75 × 10^4^ cells ml^−1,^ respectively; Fig. 5c, Fig. 6, and Fig. S5b). These cells displayed different shapes and were smaller than those detected in the epilimnion.

10.1128/mSystems.01241-20.2 FIG S2 Map of six stations on Římov Reservoir used for sampling of CARD-FISH. Different stations are color coded, and the exact coordinates of these stations are provided. Download FIG S2, PDF file, 0.08 MB. Copyright © 2021 Mujakić et al. 2021 Mujakić et al. https://creativecommons.org/licenses/by/4.0/ This content is distributed under the terms of the Creative Commons Attribution 4.0 International license.

10.1128/mSystems.01241-20.3 FIG S3 Maximum-likelihood phylogeny of the large subunit of RuBisCO (types I to III) and RuBisCO-like protein sequences (*n* = 164) of bacterial and archaeal taxa (86). The branches belonging to *Gemmatimonadota* are colored in orange. Eight freshwater MAGs of *Gemmatimonadota* contain type IV RuBisCO. The figure’s colored insets (upper left for Římov Reservoir MAGs and lower right for sediment MAGs [57]) show the MAG identifiers. The strength of support for internal nodes (assessed by ultrafast bootstrapping) is shown through colored circles (center legend). Download FIG S3, PDF file, 0.04 MB. Copyright © 2021 Mujakić et al. 2021 Mujakić et al. https://creativecommons.org/licenses/by/4.0/ This content is distributed under the terms of the Creative Commons Attribution 4.0 International license.

10.1128/mSystems.01241-20.4 FIG S4 Amino acid identity (AAI) for reconstructed freshwater MAGs of *Gemmatimonadota*, and three cultured representatives of the phyla (Gemmatimonas phototrophica, Gemmatimonas aurantiaca, and *Gemmatirosa kalamazoonesis*). The three photoheterotrophic groups (Pg1 to Pg3) are labeled with orange lines. Download FIG S4, PDF file, 0.1 MB. Copyright © 2021 Mujakić et al. 2021 Mujakić et al. https://creativecommons.org/licenses/by/4.0/ This content is distributed under the terms of the Creative Commons Attribution 4.0 International license.

10.1128/mSystems.01241-20.5 FIG S5 Overlay images of *Gemmatimonadetes* cells in Římov Reservoir visualized by CARD-FISH with probe Gemma_801. (a) Cells of *Gemmatimonadetes* in association with *Cyanobacteria* in Římov Reservoir. (b) Cells of *Gemmatimonadetes* associated with an aggregate in Římov Reservoir. The probe signal is displayed in green, DAPI staining in blue, and autofluorescence of *Cyanobacteria* in red. Download FIG S5, PDF file, 0.06 MB. Copyright © 2021 Mujakić et al. 2021 Mujakić et al. https://creativecommons.org/licenses/by/4.0/ This content is distributed under the terms of the Creative Commons Attribution 4.0 International license.

## DISCUSSION

In the presented work, we clearly demonstrated that *Gemmatimonadota* were permanently present in all of the studied lakes showing the ubiquitous nature of this phylum in contrasting freshwater lakes ranging from ultraoligotrophic to mesoeutrophic. The freshwater *Gemmatimonadota* community followed seasonal patterns with water temperature as the main driving variable, especially in the epilimnion. This observation further supports the idea that the studied *Gemmatimonadota* are truly limnic, since abundances of passively transferred microorganisms correlate with water mass movements rather than with temperature (42). The stratification of the lake also seemed to have a significant effect on the *Gemmatimonadota* community, as clusters inhabiting the epilimnion differed from those present in the hypolimnion, suggesting that they can be metabolically diverse and be specialized in different ecological roles. While *Gemmatimonadota* were relatively stable in the hypolimnion, their numbers in the epilimnion varied with higher numbers in late summer and autumn and lower numbers in spring and early summer. The stratification effect was previously studied in Grand Lake (OK, USA) where the *Gemmatimonadota* community was present in late summer in the free-living fraction within the thermocline and hypolimnion. The highest abundance in the hypolimnion at this time seemed to be associated with the sedimentation of organic matter, turbidity, and a lack of oxygen that developed in the thermocline and hypolimnion (43). The *Gemmatimonadota* community in Grand Lake was mostly composed of the genus *Gemmatimonas*, which is suggested to participate in the degradation of organic matter after an algal bloom lysis (44). In contrast, we observed that the genus *Gemmatimonas* was present in both the epilimnion and hypolimnion, but it appeared with higher abundances in the epilimnion, while the hypolimnion community was dominated by other clusters, including phototrophic group 1. However, we could also correlate the highest abundance of the genus *Gemmatimonas* in the epilimnion (15-Aug-16) with the highest abundance of the cyanobacterial community in the same metagenome sample (41). The prokaryotic community of metagenomes from Římov Reservoir was analyzed previously (41) showing *Actinobacteria* as persistently abundant in Římov Reservoir through all seasons and in both the epilimnion and hypolimnion. Other more abundant groups in the epilimnion and the hypolimnion were *Alphaproteobacteria*, *Bacteroidetes*, and *Burkholderiales* (previously *Betaproteobacteriales*). Instead, while *Cyanobacteria* were abundant in the epilimnion, they were recovered in smaller numbers, which the authors attribute to the method of sampling where most of the filamentous *Cyanobacteria* are removed.

The clear difference between samples from the epilimnion and hypolimnion was also observed using epifluorescence microscopy. The highest number of small-sized hybridized cells was detected in the hypolimnion of Římov Reservoir. In contrast, the samples from the epilimnion contained many *Gemmatimonadota* cells attached or associated with photosynthetic organisms: diatoms (*Fragilaria* sp.) or cyanobacteria (*Microcystis* sp.) (Fig. 5a and b and Fig. 6). Since both phototrophic and heterotrophic *Gemmatimonadota* require an organic source of carbon, they may benefit from the input of organic carbon from algae and cyanobacteria, providing in exchange inorganic nutrients acquired through mineralizing organic substances (45). Additionally, cells from the epilimnion, where light is available, seemed to have a larger size. Some previous studies showed that aerobic anoxygenic photoheterotrophic bacteria can often increase carbon assimilation in the presence of light, which allows them to have larger cells (46, 47). Subsequently, the distribution of *Gemmatimonadota* in the epilimnion could be dependent upon the distribution and occurrence of diatoms like *Fragilaria* sp. and cyanobacteria like *Microcystis* sp., as their blooms can influence and alter bacterioplankton communities (45, 48). Likewise, this dependence is a plausible explanation for the reason why photoheterotrophic members of this phylum are proving to be difficult to culture.

The presented 45 metagenome-assembled genomes of *Gemmatimonadota* in this study represent the largest collection of freshwater metagenome-assembled genomes (MAGs) of this phylum so far available. The obtained MAGs further confirmed the limnic nature of *Gemmatimonadota*. With recovery of several MAGs that represent the same species based on AAI (with 99 or 100% similarity), we show that *Gemmatimonadota* MAGs are reassembled from metagenomes and recovered periodically from different years as well as different seasons. This confirms the strong resilience of their microdiversity in freshwater and cannot be taken as a random occurrence. Furthermore, 39 of the obtained MAGs are different from soil species (Fig. 1; also see Fig. S1 and S4 in the supplemental material) and form distinct phylogenetic groups (with AAI between 45 and 65%) with both photoheterotrophic and heterotrophic representatives (38). Six MAGs, gained from the hypolimnion of deep lakes, three from Lake Constance (CH-Jul18-bin44, CH-Jul18-bin76, and CH-Jul18-bin112), two from Lake Zurich (ZH-3nov15-207 and ZH-3nov15-212), and one from Lake Thun (TH-Jun18-bin75), (Fig. 1 and Fig. S1) are more closely related to genomes from soil and sediments. In the 16S rRNA phylogeny (Fig. S1), two previously mentioned MAGs (CH-Jul18-bin112 and ZH-3nov15-212) clustered within the BD2-11 terrestrial group based on SILVA SSU v138 database taxonomy (12). This is consistent with 16S rRNA gene abundance in Swiss lakes where we could episodically observe the occurrence of 16S rRNA sequences related with terrestrial groups, such as *Longimicrobia*, BD2-11, and S0134 that has, as yet, been connected only with soil environments (10). Environmental 16S rRNA gene sequences from freshwater have until now been associated only with the class *Gemmatimonadetes* (10–12), and all other groups were formed with soil and sediment representatives. While the six MAGs could represent new freshwater members of the soil-connected groups, due to the close phylogenomic similarity with soil representatives and not with other freshwater MAGs, it is more probable that they represent genomes recovered due to soil runoff. Nevertheless, the MAGs present in these different phylogenetic groups were assembled from metagenomes gained from different sampling times which shows *Gemmatimonadota* were recovered repeatedly from freshwater environments. All this evidence shows that at least 39 of the identified MAGs represent truly limnic and planktonic species that do not come from soils. Furthermore, with the notable genomic diversity of limnic *Gemmatimonadota*, we demonstrate the ecological relevance of this group, as different members are clearly able to persist in the water column, occupying different ecological niches as they occur both in the hypolimnion and in the epilimnion. Moreover, the distribution of *Gemmatimonadota* in contrasting freshwater lakes showed they are adapted to different types of aquatic environments. The highest diversity in terms of different genus level groups or species of *Gemmatimonadota*, especially photoheterotrophic representatives, was observed in Římov Reservoir. Despite this, we cannot associate the higher diversity to mesoeutrophic lakes, as the data set obtained from Římov Reservoir was substantially larger, allowing for an increased chance of recovering higher diversity. A higher trophic status of any lake is connected with higher phytoplankton productivity; therefore, lakes and reservoirs often show variations of microbial communities connected with phytoplankton productivity (49, 50). Specifically, the connection of bacterial communities with changes in phytoplankton has already been recorded in Římov Reservoir (51). Furthermore, anoxygenic phototrophic bacteria are often found in close association with algae (45, 52, 53), and since they seem to follow seasonal phytoplankton blooms in freshwater lakes (45, 48), it is suggested that they represent an important functional group in freshwater environments (54).

Phototrophic gene, phylogenomic, and AAI analyses have shown that phototrophy in the *Gemmatimonadota* spans multiple genera. The identified phototrophic *Gemmatimonadota* represent three different groups but share the same set of phototrophic genes with *G. phototrophica.* All identified PGCs share a very similar gene inventory (see Table S4 in the supplemental material) and organization (Fig. 3). It seems that the convergent orientation (→ ←) of superoperons *bchFNBHLM* and *crtFbchCXYZ-puf* is conserved among all the phototrophic *Gemmatimonadota*, in contrast to phototrophic *Proteobacteria*, where orientation of these superoperons may be divergent, convergent, or colinear (55). Moreover, the split of the PGC with inserted genes in the type strain *G. phototrophica* AP64 (14) is also present in several MAGs from the epilimnion. Previously, the PGC of *G. phototrophica* was compared with two PGCs reconstructed from the Odense wastewater metagenome (OdenseWW) and the Aalborg wastewater metagenome 2 (AalborgWW-2) which did not contain the insert of several hypothetical genes between two operons. The explanation proposed for the difference in having a split PGC or not was that it could represent different evolutionary history of phototrophic *Gemmatimonadota* originating from different environments (21). Apart from the split PGC present in some of the MAGs, we observed that members of phototrophic group 1 have coenzyme F420-reducing hydrogenase (*frhB* gene) instead of bacteriochlorophyll synthase 4.5-kDa chain (*bch2*), as part of the *bchP2G* operon. Coenzyme F420-reducing hydrogenase enzyme seems to have an important role in energy conservation and methanogenesis from CO_2_ (56).

In support of the true photoheterotrophic nature of *Gemmatimonadota*, a previous study conducted in Římov Reservoir, found active expression of their *pufM* genes (a common molecular marker gene for aerobic anoxygenic phototrophs) (27). Expression of the photosynthetic apparatus of *Gemmatimonadota* showed that they are an active part of bacterial community and do not just passively contain the phototrophic genes (27).

Recently, several *Gemmatimonadota* MAGs that originate from sediments of a soda lake were reported to contain both phototrophic genes and genes related to the large subunit of ribulose 1,5-bisphosphate carboxylase/oxygenase enzyme (57), suggesting that these soda lake MAGs represent the first photoautotrophic *Gemmatimonadota* (58). In contrast, some of our freshwater *Gemmatimonadota* contain phototrophic genes as well as type IV RuBisCO (Fig. S3), which is considered only a homologue of RuBisCO, since it does not have any carboxylation activity (59, 60). Type IV RuBisCO genes are present in many microorganisms, including both phototrophic and heterotrophic bacteria and *Archaea* and are thought to participate in some other metabolic pathways different from the Calvin-Benson cycle (61, 62). Therefore, these freshwater *Gemmatimonadota* MAGs are not photoautotrophs, rather have a photoheterotrophic metabolism, typical for aerobic anoxygenic photoheterotrophic bacteria. These bacteria do not fix inorganic carbon and need to rely on organic carbon produced by other organisms, so the ability to harvest light is used to supply energy for their mostly organoheterotrophic metabolism (40, 63, 64).

In conclusion, with MAGs from these contrasting freshwater lakes, we not only reveal the existence of several new phototrophic species that differ phylogenetically from the already cultured and characterized *G. phototrophica* but also show the considerable diversity of both photoheterotrophic and heterotrophic *Gemmatimonadota* in freshwater.

## MATERIALS AND METHODS

### Sampling for metagenomics analysis.

Samples from five European freshwater lakes representing a large range of size, depth, and/or trophic status (Table 1) were used to obtain genomic information from *Gemmatimonadota*. Samples were taken from the surface mixed-sun exposed layer known as the epilimnion and the deeper, colder layer where typically there is reduced turbulence and a smaller amount of light is present. This deeper layer is called the hypolimnion. Římov Reservoir is a mesoeutrophic, canyon-shaped dimictic water body that was built during 1970s by damming a 13.5-km-long section of the River Malše (65). The sampling was performed between June 2015 and August 2017, above the deepest point of the reservoir by using a Friedinger sampler. A volume of 20 liters of water was collected from both the epilimnion (0.5 m; *n *= 10) and hypolimnion (30 m; *n *= 8) and subjected to sequential peristaltic filtration through a series of 20-, 5-, and 0.2-μm-pore-size polycarbonate membrane filters (142 mm diameter) (Sterlitech Corporation, USA). Characteristics of the water column, depth, temperature, oxygen (GRYF XBQ4; Havlíčkův Brod, Czechia) and chlorophyll *a* (FluoroProbe TS-16-12; bbe Moldaenke, Kiel, Germany) were also measured. The sample collection and filtration steps were identical for the rest of the lakes unless otherwise stated. Jiřická pond is a dystrophic humic water body from which five epilimnion (0.5-m depth) water samples were collected between May 2016 and August 2017. Lake Zurich is an oligomesotrophic, perialpine monomictic water body, from which nine water samples were collected in a period between 2010 and 2018 from the epilimnion (5-m depth; *n* = 5) and hypolimnion (80/120-m depth; *n* = 4) of the lake and processed as described above. Lake Thun is an ultraoligotrophic, alpine water body. Two water samples were collected in June 2018 from 5-m and 180-m depth. Lake Constance is a large oligomesotrophic, perialpine lake. Four samples, which were collected in July and October 2018 from the epilimnion (5 m) and hypolimnion (200 m) were used for this study.

### DNA extraction, sequencing, and assembly.

DNA was extracted from the 0.22-μm filters (0.2- to 5-μm fraction) using the ZR Soil Microbe DNA MiniPrep kit (Zymo Research, Irvine, CA, USA) in accordance with the manufacturer’s instructions. The total quantity of DNA was estimated using the Qubit dsDNA BR assay kit (Life Technologies, Foster City, CA, USA) on a Qubit 2.0 fluorometer (Life Technologies). DNA integrity was assessed by agarose gel (2%) electrophoresis and SYBR green I staining. Shotgun sequencing was performed using the Novaseq 6000 sequencing platform (2 × 150 bp) (Novogene, Hong Kong, China). Raw Illumina metagenomic reads were quality filtered in order to remove low-quality bases/reads and adapter sequences using bbmap package (66, 67). Briefly, the paired-end (PE) reads were interleaved by reformat.sh and quality trimmed by bbduk.sh (qtri = rltrimq = 18) (68). Subsequently, bbduk.sh (68) was used for adapter trimming and identification/removal of possible PhiX and p-Fosil2 contamination (k = 21 ref = vector file ordered cardinality). Additional checks (i.e., *de novo* adapter identification with bbmerge.sh [67]) were performed in order to ensure that the data sets met the quality threshold necessary for assembly. The obtained quality-filtered data sets were then assembled independently with MEGAHIT (v1.1.5) (69) using the k-mer sizes: 39, 49, 69, 89, 109, 129, 149, and default parameters.

### 16S rRNA abundance-based taxonomic classification.

The obtained quality-filtered data sets were converted to FASTA format and randomly subsampled to 20 million reads by using reformat.sh (68). These subsets (containing 20 million sequences each) were queried against the SILVA SSU database, release 132 (70), in order to identify RNA-like sequences by using MMSeqs2 (71) and an E-value cutoff of 1e−3. The bona fide 16S rRNA gene sequences (as identified by SSU-ALIGN [72]) were further compared by blastn, in nucleotide space (using as cutoff the E value 1e−5), against the SILVA SSU database amended with 16S rRNA genes recovered from *Gemmatimonadota* MAGs (see below), and classified if the sequence identity was ≥80% and the alignment length was ≥90 bp (sequences failing these thresholds were not used for downstream analyses). The taxonomic affiliation of each identified 16S rRNA read was inferred based on its best blastn hit. The relative abundances of *Gemmatimonadota* taxonomic categories were calculated as a percentage of total 16S rRNA reads.

The statistical relationships between environmental data (oxygen, temperature, and chlorophyll *a*) (41) and *Gemmatimonadota* abundance in Římov Reservoir was analyzed by distance-based linear models and nonmetric multidimensional scaling (nMDS) in the PERMANOVA+ add-on package of the PRIMER7 software (Primer Ltd., Lutton, UK). Abundance data were square root transformed, and analysis was done using a stepwise selection procedure. The best model was selected based on statistical significance (9,999 permutations), and the value of the Akaike’s information criterion (AICc) (73, 74). The same analysis was also done separately for the epilimnion and the hypolimnion.

### Recovery of bacterial genomes.

Quality-filtered metagenomics data sets were mapped using bbwrap.sh (75) (kfilter = 31 subfilter = 15 maxindel = 80) against the assembled contigs (longer than 3 kb) in a lake-dependent fashion. The resulting BAM files were used to generate contig abundance files with jgi_summarize_bam_contig_depths (--percentIdentity 97) (76). The contigs and their abundance files were used for binning with MetaBAT2 (default settings) (76). Bin completeness, contamination, and strain heterogeneity were estimated using CheckM (with default parameters) (77). Bins with estimated completeness above 40% and contamination below 5% were denominated as metagenome-assembled genomes (MAGs). MAGs were taxonomically classified with GTDB-Tk (2) using default settings.

### Phylogenomics.

MAGs belonging to *Gemmatimonadota* (*n *= 45), together with reference genomes (*n *= 226) recovered from public repositories (see Table S1 in the supplemental material) were annotated using the TIGRFAMs database (78). A total of 121 conserved marker proteins (Table S2) were extracted from the annotated *Gemmatimonadota* genomes. MAGs that had more than 40% markers present, together with reference genomes were used for phylogenetic reconstruction. Briefly, homologous proteins were independently aligned with PRANK (default settings) (79), trimmed with BMGE (-t AA -g 0.5 -b 3 -m BLOSUM30) (80), and concatenated. A maximum-likelihood phylogeny was constructed using IQ-TREE (81) with the LG+F+R10 substitution model (chosen as the best-fitting model by ModelFinder [82]) and 1000 ultrafast bootstrap replicates. Genomes appertaining to *Fibrobacterota* were used as an outgroup to root the tree.

10.1128/mSystems.01241-20.7 TABLE S2 List of 121 conserved marker proteins, which were extracted from the annotated *Gemmatimonadota* genomes and used to reconstruct the maximum-likelihood phylogeny. Download Table S2, XLSX file, 0.01 MB. Copyright © 2021 Mujakić et al. 2021 Mujakić et al. https://creativecommons.org/licenses/by/4.0/ This content is distributed under the terms of the Creative Commons Attribution 4.0 International license.

The average amino acid identity (AAI) within coherent phylogenomic groups was determined by performing whole-genome pairwise coding DNA sequence (CDS) comparison, using BLAST, as previously described (83). Taxonomic categories for the MAGs were defined using the suggested standards (38).

The photosynthetic gene clusters within obtained phylogenomic groups were analyzed in Geneious Prime 2019.2.3. For each group, MAGs with the most complete photosynthetic gene cluster were chosen as representatives. The alignment and identity matrix for nucleotide *pufM* and *acsF* gene sequences from photoheterotrophic MAGs and *G. photorophica* was done with ClustalW 2.0.10 (84).

### Phylogenetics.

The 16S rRNA sequences present in the recovered MAGs were identified by SSU-ALIGN (72). The ones with a length longer than 200 nucleotides (nt) were merged with a data set comprising *Gemmatimonadota* sequences recovered from SILVA SSU v138 database (reference NR 99; length, 1200 bp) (70). The sequences were aligned with PASTA v1.8.3 (85) and used to construct a maximum-likelihood phylogeny with IQ-TREE v1.6.10 (-m GTR+F+R10; chosen as the best fitting model by ModelFinder) (81, 82). Several sequences appertaining to *Cyanobacteria* and *Fibrobacterota* were used as outgroup to root the tree.

Eighteen proteins belonging to the large subunit of the ribulose 1,5-bisphosphate carboxylase enzyme (RuBisCO) were identified in the assembled MAGs (*n *= 106). These proteins, together with a data set comprised of 146 RuBisCO (types I to III) and RuBisCO-like (type IV) proteins (86) were treated with PREQUAL v1.02 (87) prior to alignment with PASTA v1.8.3 (85). The obtained alignment (854 aligned positions) was used to construct a maximum-likelihood phylogeny with IQ-TREE v.1.6.10 and the LG+F+R6 substitution model (chosen as the best-fitting model by ModelFinder) (81, 82).

### Fragment recruitment.

The obtained MAGs and the metagenomic data sets were used to compute genome coverage per gigabase using RazerS 3 (using cutoffs of >95% identity and alignment lengths of ≥50 bp) (88). All rRNA sequences (5S, 16S, and 23S) present in the MAGs were identified using rrna_hmm (89) and were masked prior to comparisons with quality-filtered metagenomic sequences. The quality-filtered data sets were used to compute abundance profiles for the *Gemmatimonadota* MAGs. Raw data showing coverage per gigabase is shown in Table S3, and a heatmap (Fig. 4) was created using http://heatmapper.ca.

10.1128/mSystems.01241-20.8 TABLE S3 Recruitment values of 27 *Gemmatimonadota* MAGs obtained from Římov Reservoir in 28 metagenomes from Římov Reservoir. Values represent genome coverage per gigabase. Download Table S3, XLSX file, 0.01 MB. Copyright © 2021 Mujakić et al. 2021 Mujakić et al. https://creativecommons.org/licenses/by/4.0/ This content is distributed under the terms of the Creative Commons Attribution 4.0 International license.

10.1128/mSystems.01241-20.9 TABLE S4 List of phototrophic genes found in Gemmatimonas phototrophica and photoheterotrophic MAGs of *Gemmatimonadota*. The first sheet presents names of all phototrophic genes present in MAGs, and the second sheet contains detailed information and exact list of all phototrophic genes found in each MAG as well as their placement on individual contigs. Download Table S4, XLSX file, 0.02 MB. Copyright © 2021 Mujakić et al. 2021 Mujakić et al. https://creativecommons.org/licenses/by/4.0/ This content is distributed under the terms of the Creative Commons Attribution 4.0 International license.

### Design of oligonucleotide probes.

We designed a new oligonucleotide probe that targets a part of the class *Gemmatimonadetes* (Fig. S1). Originally, the probe was designed as a *Gemmatimonadetes*-specific broad-range (degenerate) PCR primer. *Gemmatimonadetes* and non-*Gemmatimonadetes* 16S rRNA gene sequences were retrieved from SSURef SILVA database (release 132 [70]), and aligned with the MUSCLE60 algorithm in MEGA7 software (90). The primers were then designed manually to target conserved regions specific for *Gemmatimonadetes*. The specificity of the primers was first tested *in silico* with the SILVA database (70) and then with PCR using DNA from Gemmatimonas phototrophica which was used as a positive control. Optimized PCR conditions were as follows: initial denaturation at 98°C for 3 min; 26 cycles with 1 cycle consisting of 98°C for 10 s, 60°C for 20 s, and 72°C for 20 s; and final elongation for 3 min. Primers were additionally tested with several other bacterial strains, which served as negative controls and showed no amplification. Finally, the primers were used on an environmental sample where their specificity was confirmed with Illumina amplicon sequencing. Briefly, three different primer sets were applied to an environmental sample collected at station DAM of Římov Reservoir. After Illumina sequencing, read quality was evaluated using FastQC v0.11.7 (Babraham Bioinformatics, Cambridge, UK). Primer sequences were trimmed using cutadapt v1.16 (91) and subsequently analyzed in the R/Bioconductor environment using the dada2 v1.6 package (37). Taxonomic assignment was performed using the SILVA 132 database (70). On the basis of these results, we opted to use the reverse primer (5′ TCG CTC CCC CAR RSA CCT AGT 3′) as a CARD-FISH-probe Gemma_801. As the probe-binding site was located within a hairpin loop and the accessibility of the binding site was low (92), we designed two 18-bp-long helpers to open the loop and facilitate hybridization (93): Gemma_801 H1 (5′-GCG CCG GCA YYC GAG GGG-3′) and Gemma_801 H2 (5′-GGG GDA CTT AAT GCG TTA-3′). The specificity of the probe was tested *in silico* using the ARB Probe_Match function and the SILVA online ProbeCheck tool against the ENA database (EMBL-EBI). The probe matches only six untargeted sequences and six more if one weighted mismatch is allowed (<0.0021% of all bacterial sequences), and all of them originate from soil or the rhizosphere. The coverage of the probe for class *Gemmatimonadetes* is 35%, so the usage of this probe was primarily justified with the intent to visualize the *Gemmatimonadetes* cells in the natural environment, and not to use it for absolute quantification. Hybridization conditions were optimized at 35°C and 46°C by a stepwise increase of formamide concentrations in the hybridization buffer (20% to 70% by 5%) using pure cultures of *G. phototrophica* AP64 and photoheterotrophic strain TET16 (36) as positive controls, and Limnohabitans planktonicus (*Gammaproteobacteria/Betaproteobacteriales*) and *Sphingomonas* sp. strain AAP5 (*Alphaproteobacteria*) as negative controls. The final protocol is described below.

### Sampling for catalyzed reporter deposition-fluorescence *in situ* hybridization (CARD-FISH) analysis.

We applied the newly designed probe on a sample set collected between 14 May and 24 August 2015 at Římov Reservoir. Six permanent stations (river, stations 2, 3, 4, and 7, and dam) along the longitudinal transect of the reservoir (94) (Fig. S2) were sampled with a Friedinger sampler at 3-week intervals. Samples were taken from 0.5-m depth at all stations and at additional depths at lacustrine stations: 5 m and 10 m at stations 4 and 7; and 5 m, 10 m, 20 m, 30 m, and 40 m at the dam site. Water samples were fixed on-site with formaldehyde (2% final concentration [vol/vol]) and transported to the laboratory within 2 h. For the enumeration of bacterial densities,1 to 2 ml was stained with 4′,6′-diamidino-2-phenylindole (DAPI) and filtered onto black 0.2-μm-pore-size polycarbonate membranes (25 mm diameter; SPI Supplies, USA), and bacterial abundance was determined via epifluorescence microscopy (95).

### CARD-FISH analysis.

Fixed formaldehyde water samples containing 1 × 10^6^ to 2 × 10^6^ bacteria were filtered onto polycarbonate membranes with a 0.2-μm pore size (47-mm diameter; Millipore, USA), and filters were stored at −20°C. The catalyzed reporter deposition-fluorescence *in situ* hybridization (CARD-FISH) analysis was conducted as in the previously described protocol (96) with slight modification in digestion procedure, optimized for samples from Římov Reservoir. In brief, the microorganisms were immobilized on the membranes with low-melting-point agarose and permeabilized with lysozyme (10 μg ml^−1^, 1 h) and achromopeptidase (60 U ml^−1^, 20 to 25 min). Digestion was followed by neutralization of endogenous peroxidases and hybridization with the horseradish peroxidase-labeled probe Gemma_801 (biomers.net GmbH) and helpers in a hybridization buffer with 35% formamide concentration (vol/vol) at 35°C for 3 h. After the subsequent washing steps, amplification of the hybridization signal was performed with fluorescein-labeled tyramides (Invitrogen, Carlsbad, CA, USA) at 37°C for 30 min. Washed and dried filters were counterstained with DAPI (1 μg ml^−1^) and analyzed with epifluorescence microscopy (Olympus BX-53F) using UNWU, U-WB, and U-WG optical filter sets. Proportions of CARD-FISH stained bacteria were determined by inspecting more than 1000 DAPI-stained cells per sample.

### Data availability.

Sequence data for all metagenomes generated in this work are archived at EBI European Nucleotide Archive and can be accessed under the BioProject accession number PRJEB35770. All metagenome-assembled genomes are also available at figshare (https://doi.org/10.6084/m9.figshare.12662132.v2).
