# Supplementary figures and images for: Common Presence of Phototrophic Gemmatimonadota in Temperate Freshwater Lakes
Source: mSystems. 2021 Mar 16;6(2):e01241-20. doi: 10.1128/mSystems.01241-20 (PMC8547001; doi:10.1128/mSystems.01241-20)

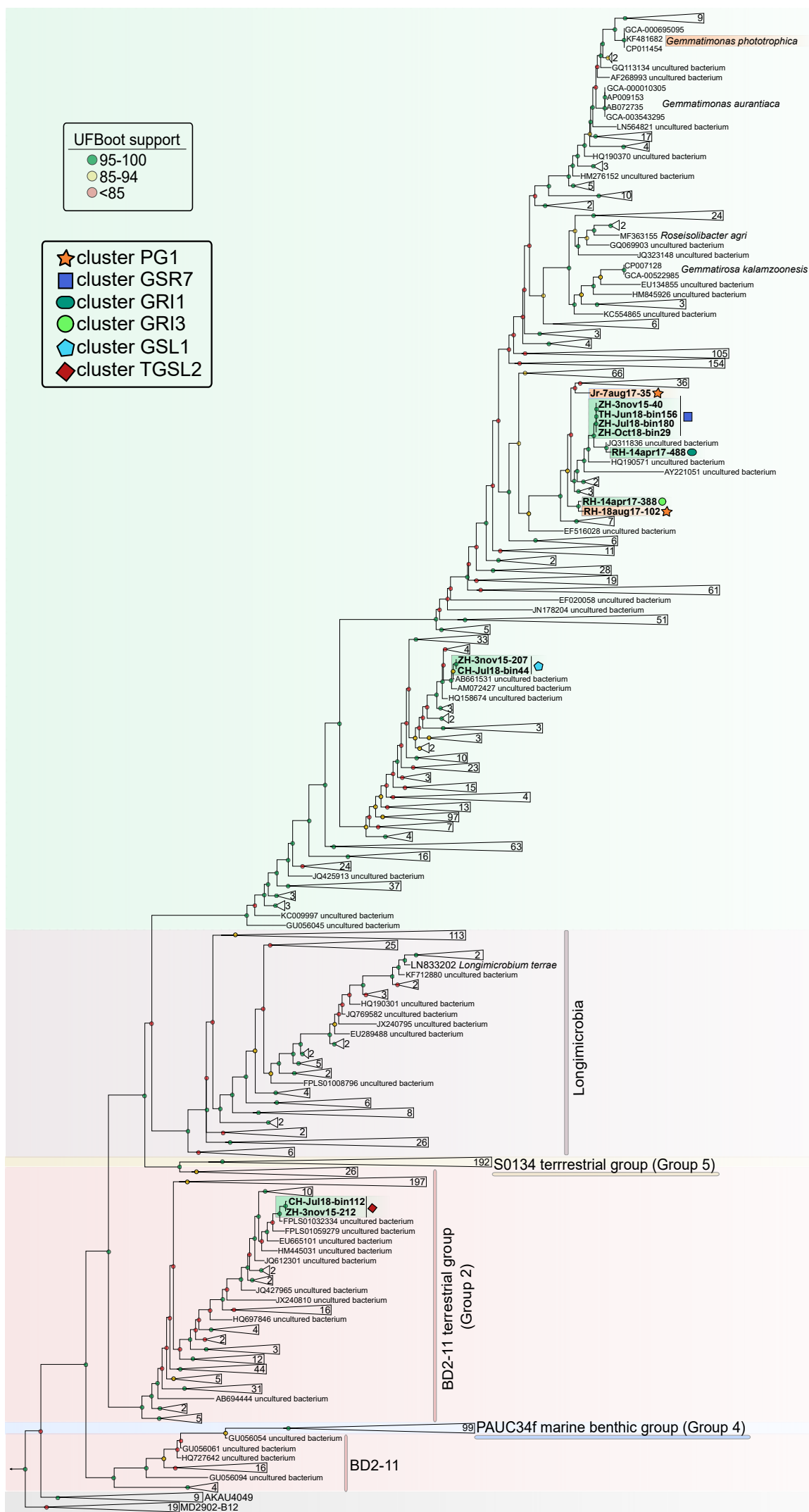

Supplement: FIG S1 [file msystems.01241-20-sf001.pdf]

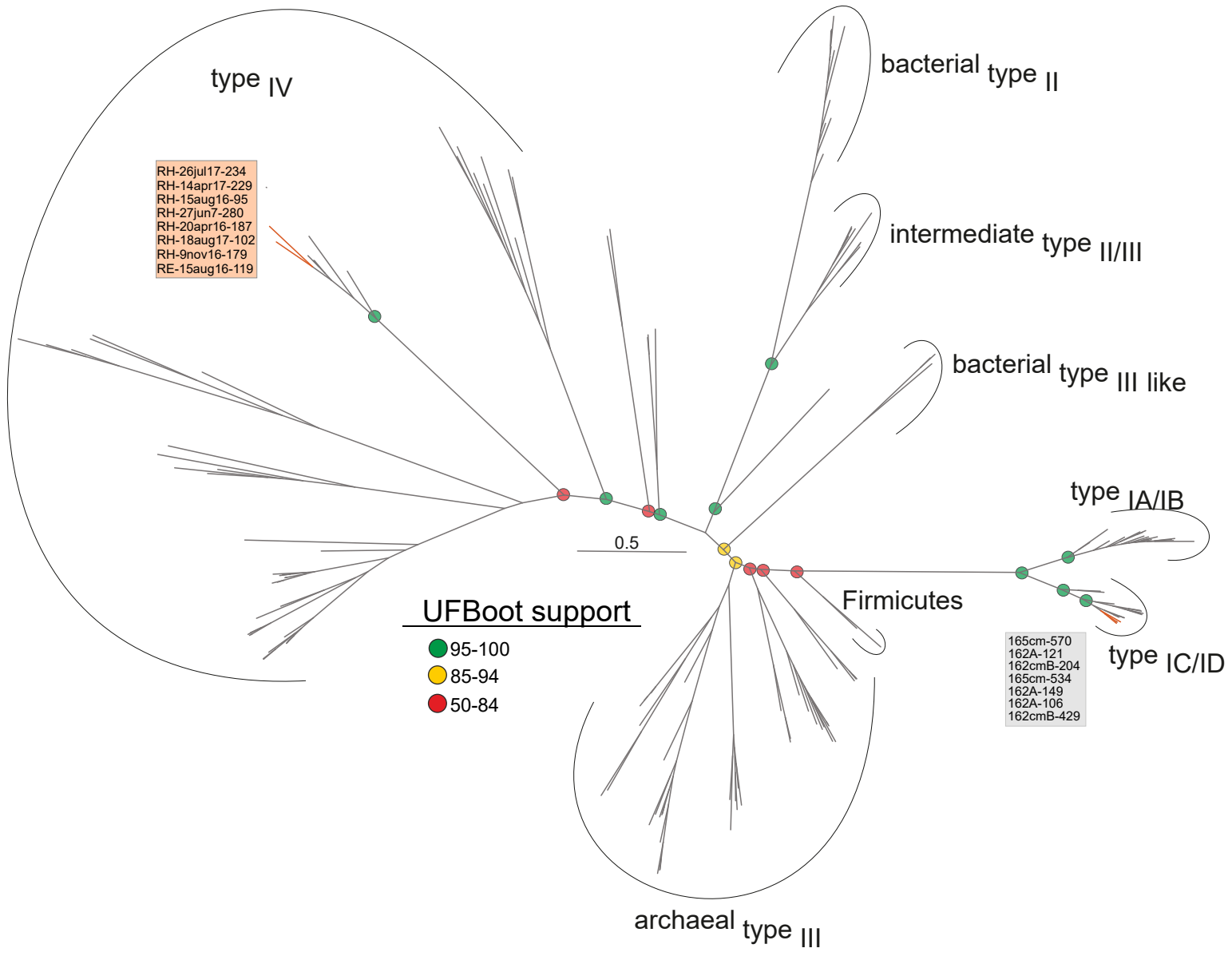

Supplement: FIG S3 [file msystems.01241-20-sf002.pdf]

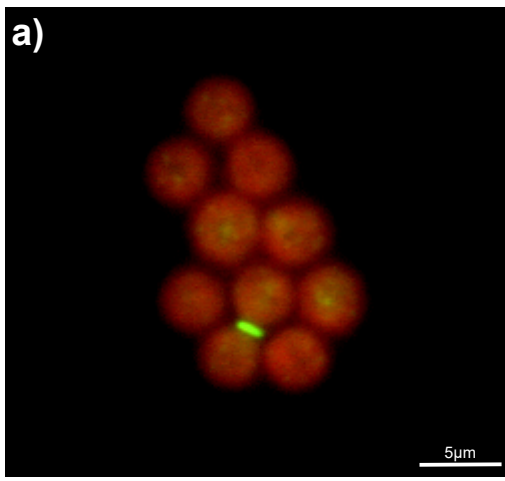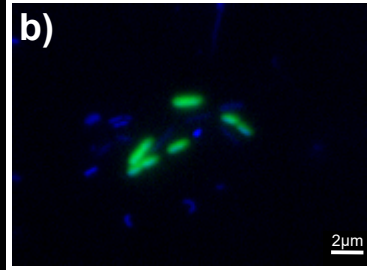

Supplement: FIG S5 [file msystems.01241-20-sf005.pdf]
