# Supplementary material for: Common Presence of Phototrophic Gemmatimonadota in Temperate Freshwater Lakes
Source: mSystems. 2021 Mar 16;6(2):e01241-20. doi: 10.1128/mSystems.01241-20 (PMC8547001; doi:10.1128/mSystems.01241-20)
Supplement: FIG S2 [file msystems.01241-20-sf004.pdf]

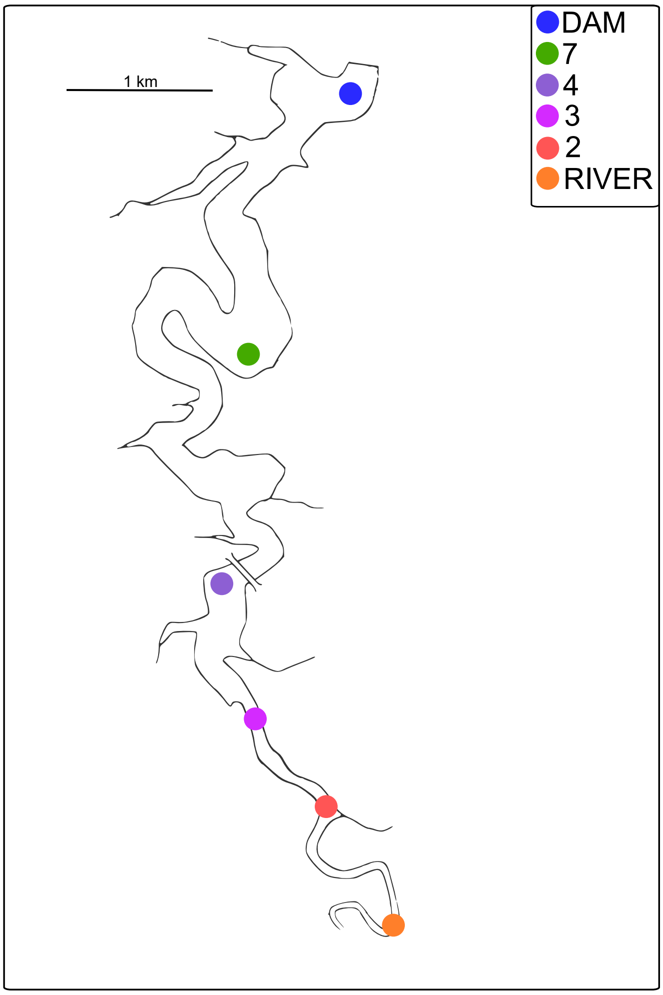

SAMPLING POINT	COORDINATES
DAM	48.8475817N, 14.4902242E
7	48.8325119N, 14.4833283E
4	48.8205219N, 14.4819550E
3	48.8136697N, 14.4840028E
2	48.8072928N, 14.4920892E
RIVER*	48.8009589N, 14.4979022E

*inflow, above the plunge point, position changes according to water level; water level 468- 469 m a.s.l.
