# Supplementary material for: Common Presence of Phototrophic Gemmatimonadota in Temperate Freshwater Lakes
Source: mSystems. 2021 Mar 16;6(2):e01241-20. doi: 10.1128/mSystems.01241-20 (PMC8547001; doi:10.1128/mSystems.01241-20)
Supplement: FIG S4 [file msystems.01241-20-sf003.pdf]

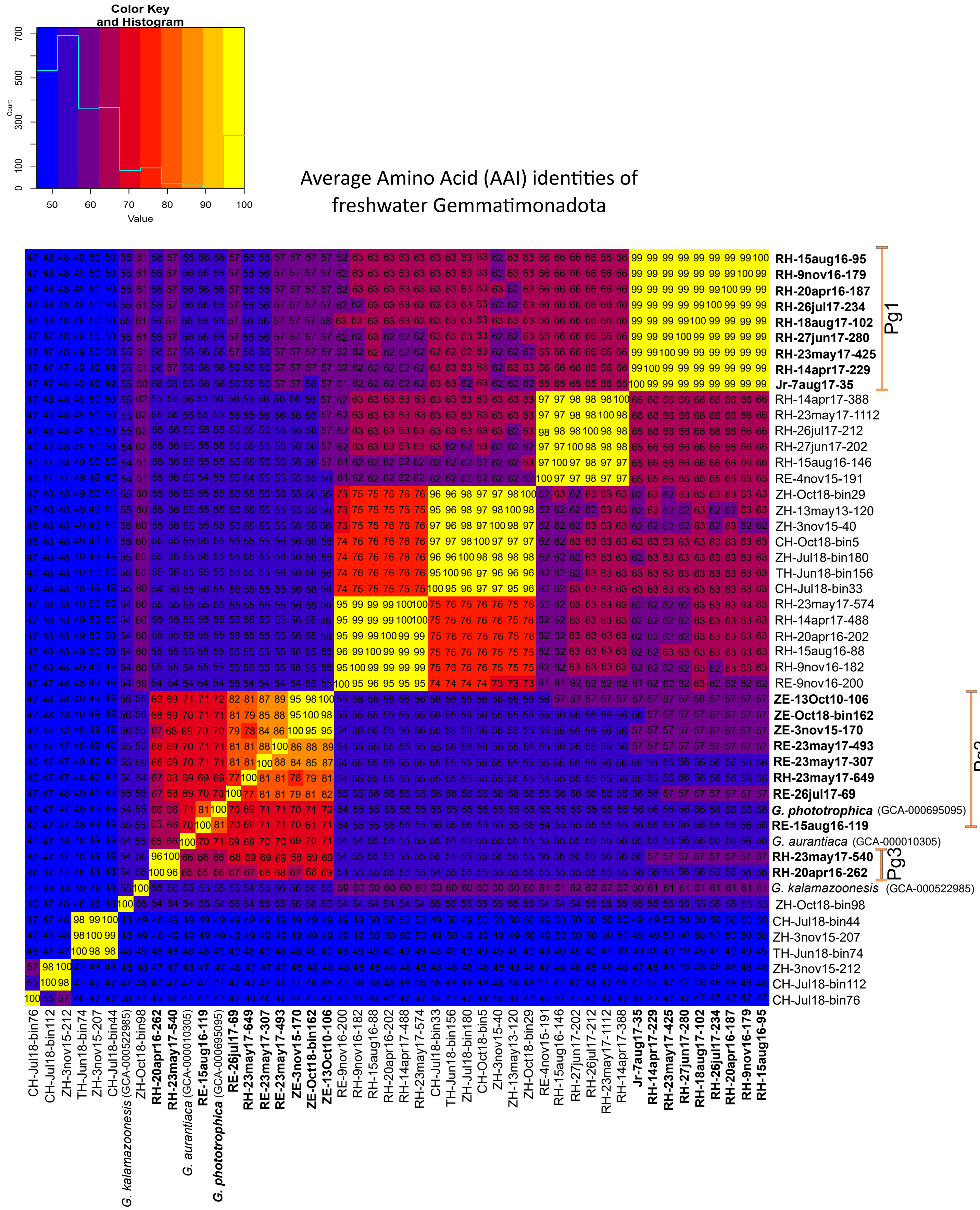
Color Key and Histogram
Count
Value
Average Amino Acid (AAI) identities of freshwater Gemmatimonadota
Pg1
Pg2
Pg3
RH-15aug16-95
RH-9nov16-179
RH-20apr16-187
RH-26jul17-234
RH-18aug17-102
RH-27jun17-280
RH-23may17-425
RH-14apr17-229
Jr-7aug17-35
RH-14apr17-388
RH-23may17-1112
RH-26jul17-212
RH-27jun17-202
RH-15aug16-146
RE-4nov15-191
ZH-Oct18-bin29
ZH-13may13-120
ZH-3nov15-40
CH-Oct18-bin5
ZH-Jul18-bin180
TH-Jun18-bin156
CH-Jul18-bin33
RH-23may17-574
RH-14apr17-488
RH-20apr16-202
RH-15aug16-88
RH-9nov16-182
RE-9nov16-200
ZE-13Oct10-106
ZE-Oct18-bin162
ZE-3nov15-170
RE-23may17-493
RE-23may17-307
RH-23may17-649
RE-26jul17-69
G. phototrophica (GCA-000695095)
RE-15aug16-119
G. aurantiaca (GCA-000010305)
RH-23may17-540
RH-20apr16-262
G. kalamazoonesis (GCA-000522985)
ZH-Oct18-bin98
CH-Jul18-bin44
ZH-3nov15-207
TH-Jun18-bin74
ZH-3nov15-212
CH-Jul18-bin112
CH-Jul18-bin76
